# IRL790 modulated striatal D1 neurons synaptic plasticity ameliorating levodopa-induced dyskinesia in mouse

**DOI:** 10.3389/fnagi.2024.1401991

**Published:** 2024-05-30

**Authors:** Xiaofei Wang, Wangming Zhang

**Affiliations:** ^1^Zhujiang Hospital, Southern Medical University, Guangzhou, China; ^2^Guangdong Provincial Key Laboratory on Brain, Zhujiang Hospital, Southern Medical University, Guangzhou, China

**Keywords:** levodopa-induced dyskinesia, dopamine D3 receptor, structural plasticity, functional plasticity, Parkinson’s disease

## Abstract

**Objective:**

Levodopa (L-dopa) therapy is the principal pharmacological treatment for Parkinson’s disease (PD). Nevertheless, prolonged use of this drug may result in different involuntary movement symptoms caused by the medication, referred to as levodopa-induced dyskinesia (LID). LID is associated with changes in synaptic plasticity of the D1 medium spiny neurons (MSNs) located in the dorsal striatum (dStr). Within the striatum, the amount of Dopamine D3 receptor (D3R) is notably increased in LID, demonstrating colocalization with D1R expression in neurons, and the level of D3R expression is directly related to the intensity of LID. IRL 790, as a D3R antagonist, can ameliorate LID. This study aims to explore if IRL 790 improves LID by regulating the synaptic plasticity of D1+ MSNs in dStr.

**Methods:**

The electrophysiology and synaptic spine density of D1+ MSNs in dStr were recorded for sham mice, LID mice, and LID mice treated with IRL 790. The regulation of synaptic plasticity in LID D1+ MSNs by IRL 790 was analyzed. Behavioral tests were conducted to confirm the treatment effect of IRL 790 on LID.

**Results:**

In LID D1+ MSNs, there was persistent abnormal LTP, absence of LTD, and an increase in spontaneous excitatory postsynaptic currents (sEPSCs). IRL 790 treatment restored normal LTP, LTD, and sEPSCs. Treatment with IRL 790 also restored the reduced dendritic spine density in D1+ MSNs of LID mice. IRL790 improved dyskinetic manifestations in LID mice.

**Conclusion:**

IRL790 ameliorates LID by regulating the synaptic structure and functional plasticity of striatal D1+ MSNs.

## 1 Introduction

Parkinson’s disease is widely recognized as a prevalent neurological disorder that belongs to a gradually degenerative condition of the nervous system, involving motor function impairments and non-motor issues. According to research, it ranks second among neurodegenerative diseases (Ascherio and Schwarzschild, 2016). It is estimated that over 4 million people worldwide are affected by this condition, which is rare in individuals under 40 years of age, and the prevalence of the disease increases with age. Data indicates that among the elderly over the age of 80, approximately 3% are afflicted by this condition (Dexter and Jenner, 2013). Further investigations have revealed that the frequency of the disease in males is roughly twice that of females, with men typically encountering this condition 2 years earlier than women (Van Den Eeden, 2003). The main feature of PD is the progressive loss of dopaminergic neurons in the substantia nigra pars compacta (SNc) and the decrease in dopamine (DA) levels in the striatum, leading to motor impairments (Bastide et al., 2015).

L-dopa therapy is the main pharmacological treatment for PD. Nevertheless, prolonged take of this drug may result in various late-stage, drug-induced involuntary movement disorders referred to as LID. Approximately 40% of individuals with Parkinson’s disease experience LID within 4–6 years of initiating treatment, eventually impacting the majority of patients (Ahlskog and Muenter, 2001).

Current research suggests that the occurrence of LID may involve neurocircuitry remodeling characterized by pathological synaptic plasticity manifestations. In PD patients, there is significant denervation of dopaminergic neurons in the SNc and a marked reduction in dopamine levels within the dStr, leading to diminished ability to normally store and release dopamine, as well as a compromised clearance of synaptic clefts by the dopamine transporter (DAT) (Meissner et al., 2006). The prolonged intake of exogenous L-dopa leads to non-physiological dopamine fluctuations stimulating the striatal DA receptors, causing abnormal plasticity changes in the synaptic structure and function of the cortical-striatal connections (Picconi et al., 2003, 2008). The striatal dopamine receptors are divided into D1-like (D1R, D5R) and D2-like (D2R, D3R, D4R) receptors. D1-like is mainly involved in direct pathway regulation and plays a role in promoting movement. D2-like is mainly involved in indirect pathway regulation and plays an inhibitory role in motor movement. The pathogenesis of LID is considered to be closely associated with the direct pathway, especially changes in synaptic plasticity within it, including structural and functional plasticity. In terms of synaptic structure, there are abnormalities in the synaptic architecture of striatal D1+ MSNs, including an decrease in the density and changes in morphology of dendritic spines (Fieblinger et al., 2014). In terms of synaptic function, there is an abnormal and sustained Long-term potentiation (LTP) and an inability to induce Long-term depotentiation (LTD) in MSNs expressing D1R (Zhang et al., 2013).

The D3R was successfully cloned and molecularly characterized for the first time in 1990 (Sokoloff et al., 1990). Since its discovery, it has gained attention as a potential, albeit complex, target for treatments of Parkinson’s disease, drug abuse, and schizophrenia (Cortés et al., 2016; Galaj et al., 2018). D3R is part of the D2-like family of G protein-coupled receptors (GPCRs), which includes D2R and D4R, and serves as a prototype that is coupled with Gi proteins. This coupling leads to the inhibition of adenylyl cyclase (AC) signaling and its downstream effectors, in contrast to the D1-like receptor family (D1R, D5R) that is coupled with Gs/q/olf proteins and enhances downstream signaling (Beaulieu et al., 2015).

In LID, the expression and function of D3R in the striatum have changed. In 1997, Bordet and his team observed that the abnormal presence of D3R in the striatum was caused by the use of L-dopa and played a role in behavioral sensitization (Bordet et al., 1997). Subsequently, this occurrence has been duplicated in various other research facilities and representations of LID. In rats and mice treated with by 6-OHDA (Bordet et al., 2000; Farré et al., 2015; Solís et al., 2015), mice treated with MPTP (Cote and Kuzhikandathil, 2015), and monkeys treated with MPTP (Quik et al., 2000; Sánchez-Pernaute et al., 2007; Farré et al., 2015), animals with LID exhibited high D3R expression. Further research has shown that in LID, D1R, and D3R coexist in direct pathway MSNs, and there is a direct link between the two (Ridray et al., 1998). Importantly, after L-dopa administration, the level of D3R significantly increases in the striatum and shows neuronal colocalization with D1R expression (Farré et al., 2015); moreover, abundant evidence suggests a linear correlation between the expression level of the D3R and the severity of LID, making D3R the singular dopamine receptor implicated in this relationship (Bézard et al., 2003; Azkona et al., 2014). The use of selective D3R antagonists by some research groups to improve LID behaviors also suggests D3R’s involvement in the genesis and progression of LID (Hsu et al., 2004; Kumar et al., 2009; Fanni et al., 2019). In a pivotal study by Solis et al. in 2017, it was discovered that globally knocking out D3R not only mitigates LID manifestations but also decreases markers in D1+ neurons, in the context of LID. This study employed both genetic and pharmacological approaches to delineate the intricate relationship between D1R and D3R, revealing a reduction in FosB expression and histone 3 acetylation primarily in D1 MSNs (Solís et al., 2015). Such findings highlight the interconnectedness of D1R and D3R within the milieu of LID. D1R and D3R deviates from the normal physiological state, being collaborative rather than antagonistic. The role of D3R antagonists, when present during levodopa therapy, lowers direct pathway markers linked to cellular activation, suggesting a synergistic relationship in signal transduction between the two receptors (Solís et al., 2015).

Existing research into the role of D3R antagonists for the treatment of LID has primarily been from a molecular biology perspective. The regulatory effects of D3R antagonists on the synaptic structure and functional plasticity of the striatum’s direct pathway in LID remain unclear. From the text above, it is known that the pathogenesis of LID is closely related to changes in synaptic plasticity in the direct pathway D1+ MSNs, accompanied by high expression of D3R. We hypothesize that D3R antagonists improve LID manifestations by regulating the abnormal synaptic plasticity expressed by striatal D1+ MSNs. Therefore, as an approximation to that question, we utilized a D3R antagonist injected intracranially at concentrations that exert antagonism not only on D3R but also off-target activity on D2R, serotonergic, and sigma receptors, as well as norepinephrine transporter, among others (Waters et al., 2020). The results show that IRL790 evokes changes in synaptic plasticity of striatal D1+ MSNs in LID mouse models.

## 2 Materials and methods

### 2.1 Animals

The experiment used 26 male D1-Cre mice, weighing 20 grams. The subjects were kept in a room with regulated temperature (around 25°C) and a 12-h light/dark cycle, and were provided with unlimited food and water. The Institutional Animal Care and Use Committee of Southern Medical University, China approved all experiments (LAEC-2022-146). The procedures were carried out in alignment with the NIH publication No. 8023, revised 1978, titled “Guide for the Care and Use of Laboratory Animals,” as published by the U.S. National Institutes of Health.

### 2.2 Experimental procedure

**Figure 1:**
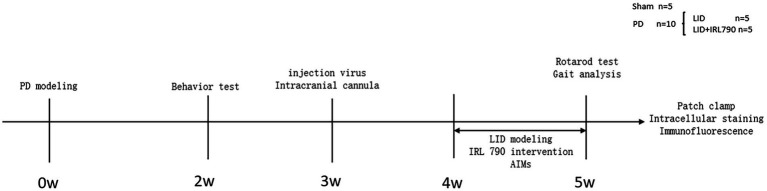
Schematic experimental protocol.

The 26 mice were numbered and divided into a PD group (*n* = 20) and a Sham group (*n* = 6). On the first day of the experiment, unilateral PD lesions were induced by injecting 6-OHDA (3 μg/μL, 1 μL, dissolved in 0.9% w/v saline) into the medial forebrain bundle (MFB) on the right side. The Sham group was injected with saline (1 μL) to induce a sham lesion. Open field and apomorphine-induced rotation tests were conducted on day 14 to assess the success of the PD model creation. Within 2 weeks after the surgery, 8 mice from the PD group died, and 1 from the sham group died. The successful creation of PD model was confirmed by the apomorphine-induced rotation test in 10 out of 12 PD group mice. The 10 successfully modeled mice were randomly divided into an LID group (*n* = 5) and an IRL790 + LID group (*n* = 5). On day 21 post-surgery, 15 mice underwent viral injection of rAAV-Ef1a-DIO-mCherry-WPRE-pA into the dStr with the purpose of labeling D1+ neurons and implantation of intracranial cannulas, followed by a week of recovery. Then, for 7 consecutive days, the IRL790 + LID group was injected intracranially with IRL790 (3.2 μL, 50 mg/mL, 71.350 mM, 500 nL/min, dissolved in 0.9% w/v saline), and the rest were injected intracranially with an equal volume of saline. 15 min after intracranial injection, the LID group and IRL790 + LID group mice were given L-dopa (12 mg/kg, 2 mg/mL, dissolved in 0.9% w/v saline) and benserazide (15 mg/kg, 3 mg/mL, dissolved in 0.9% w/v saline) by intraperitoneal injection, while the sham group was injected intraperitoneally with an equal volume of saline. The Abnormal Involuntary Movement Scale (AIMs) scoring was assessed each day after drug administration for 7 days. On day 7, rotating rod and gait analysis tests were conducted. After these experiments concluded, biochemical, electrophysiological, and intracellular staining experiments were conducted for each group (Figure 1).

### 2.3 Surgeries

For the induction of unilateral PD lesion by 6-OHDA, mice were anesthetized through intraperitoneal injection of pentobarbital sodium (50 mg/kg), then fixed in a stereotaxic instrument. The coordinates for the right MFB were determined according to “The Mouse Brain in Stereotaxic Coordinates” (Second Edition by Paxinos, G. and Franklin, K.B.J., Academic Press, New York, 2001, ISBN 0–12-547637-X): anterior to the bregma (AP) -1.2 mm, right lateral to the midline (ML) +1.2 mm, and dorsoventral (DV) -4.8 mm. At these coordinates, 6-OHDA (3 μg/μL, 1 μL, 200 nL/min, dissolved in 0.9% w/v saline) or saline was injected into the MFB with a 5 μL syringe.

For the injection of rAAV-Ef1a-DIO-mCherry-WPRE-pA (titre: 2.94 × 1012VG/ml) and the implantation of intracranial drug delivery cannula (Outside diameter: 0.64 mm, Inside diameter: 0.4 mm), mice were anesthetized through intraperitoneal injection of pentobarbital sodium (50 mg/kg), then fixed in a stereotaxic instrument. The virus rAAV-Ef1a-DIO-mCherry-WPRE-pA was injected into the right dStr of the mouse (A*P* + 0.7 mm, ML + 1.7 mm, DV-3.0 mm) according to “The Mouse Brain in Stereotaxic Coordinates” in a volume of 500 nL (200 nL/min) with a 5 μL syringe. After the virus injection, an intracranial drug delivery cannula was implanted at this same coordinate and fixed onto the skull with dental cement.

### 2.4 Behavioral tests

Prior to initiating any behavioral experiments, the mice were given a 30 min acclimation period to familiarize themselves with the experimental environment. All behavioral experiment scores were rated by an experimentally blinded investigator.

For the open field test, mouse was placed in an opaque open box measuring 40× 40× 40 cm. They were allowed to move freely in the box for 30 min. The distance traveled by the mice was assessed using a video camera and computer analysis software.

For the APO test, apomorphine was injected intraperitoneally (0.25 mg/kg) to induce rotation, and the number of rotations toward the lesion side was recorded for 5 min. An average of >6 turns/min determined a mouse as a PD model.

The rotarod test included a 3-day pre-training period, during which the mice were acclimated to the rotarod apparatus before the formal experiment. During the three-day pre-training, the mice underwent adaptation training at speeds of 6, 8, and 10 rpm, with a 20-min interval between each trial for fatigue recovery. The pre-training was conducted three times per day. In the formal experiment, the mice were placed on the rotating rod, which was uniformly accelerated from rest at an acceleration of 10 rpm, and the time the mice fell off the rotating rod was recorded. The Rotarod apparatus was provided by Beijing Zhishu Duobao Biological Technology Co., Ltd.

For the gait analysis experiment, the limbs of the animal are immersed in dye, the dyed animal is gently placed at the beginning of the recording paper, and the animal is allowed to walk freely to the other end of the paper. When an animal walks, it leaves a continuous footprint on the recording paper through dyed footprints, accurately measuring the distance between the left and right rear PAWS (a) and the distance between the same paw (left rear paw) (b) to assess the degree of parallel movement of the limbs (compare a/b ratios).

The mice were evaluated by abnormal involuntary motor score (AIMs) after l-dopa injection. Score was performed from four aspects: motor, forelimb, orolingual and axial (Lundblad et al., 2004). Score was 0–4 for each part according to severity: 0 score, no abnormal movement; 1, the time of movement disorder is less than half of the observation time; 2 points, the time of movement disorder was more than half of the observation time; 3, the dyskinesia persisted, but stopped after stimulation; 4 points, the dyskinesia persists and does not stop after stimulation. After medication, the score was scored every 20 min, and the score was observed for 2 min each time, and the score was scored for 9 times, a total of 180 min. The AIMs score for each time was the sum of the four scores for the motor, forelimb, orolingual and axial. Each day’s AIMs score is the sum of the 9 times AIMs scores on the day.

### 2.5 Electrophysiological experiments

Mice were anesthetized with pentobarbital sodium (50 mg/kg) via intraperitoneal injection within 3 h of the last injection of levodopa. Striatal brain slices for electrophysiological recordings were obtained using a vibrating microtome, with a thickness of 300 μm. The slices were then placed in artificial cerebrospinal fluid (ACSF) at around 34°C for half an hour before being moved to room temperature (22–25°C) for future use.

Throughout, the liquids were perfused with a gas mixture (95% O2 + 5% CO2) to maintain proper pH and oxygen concentration. The ACSF was composed of the following (in mmol/L, pH = 7.2, osmolarity about 280 ~ 320 mOsm): NaCl 126, KCl 2.5, CaCl2H2O 2, NaH2PO4H2O 1.25, MgCl26H2O 1, NaHCO3 26, glucose 10.

Glass electrodes with a resistance of about 3–6 MΩ were filled with an appropriate amount of pipette solution at the tip for electrophysiological recordings of striatal D1+ MSNs. The pipette solution consisted of the following (in mmol/L, pH = 7.2, osmolarity about 290 mOsm): K-Glucose 135, KCl 5, MgCl2 2, HEPES 10, Na2-ATP 0.2, Mg-GTP 2. In voltage clamp mode, the holding voltage was set to −70 mV. After waiting for cell stabilization, the protocol was adjusted to record sEPSCs, LTP, and LTD.

All signals were amplified by an Axopatch 200B microelectrode amplifier, and signal conversion was performed by an Axon Digidata 1,550 A/D converter (both from Molecular Devices, United States), with a filtering frequency of 5 kHz and a sampling frequency of 10 kHz. Signals were recorded using pClamp 10.2 software (Molecular Devices, United States) running on a computer. LTP and LTD were induced via an Spike-Timing-Dependent Plasticity (STDP) stimulation protocol.

### 2.6 Intracellular staining

After the whole-cell recording, selected D1+ MSNs were labeled with the fluorescent dye Lucifer Yellow. The dye was added to the glass electrode, which was then pierced into the cell body to enter the cell interior. Ionophoresis was used to inject Lucifer Yellow for intracellular staining of the neuron until all fine dendritic branches showed bright fluorescence (Buhl and Schlote, 1987; Buhl and Lübke, 1989). A confocal laser scanning microscope was used to obtain dendritic spine fluorescence images.

### 2.7 Immunofluorescence staining

Following the electrophysiological recording, the excess brain tissue was preserved in 4% formaldehyde for a duration of 24 h, followed by dehydration using a 30% sucrose solution. Slices, 40 micrometers thick, of the SNc were then precisely cut using a freezing microtome.

These slices were carefully placed onto a 12-well plate and thrice rinsed with PBS to ensure cleanliness. For the purpose of blocking, each well subsequently received 500 μL of PBS mixed with 5% Bovine Serum Albumin (BSA) and 1% Triton X-100, and was left to incubate at room temperature for 1.5 h. After another series of three PBS washes, the slices were incubated overnight at 4°C on a shaker. This incubation involved PBS containing rabbit anti-TH polyclonal antibody at a 1:500 dilution.

Following yet another triplet of PBS washes, the slices were exposed to 500 μL of PBS containing Alexa Fluor 594 chicken anti-rabbit IgG secondary antibody, also diluted to 1:500, for 2 h at room temperature away from light. Subsequently, after a solitary wash with PBS, the slices were treated with 500 μL of PBS mixed with DAPI (1,500) for nuclear staining, which took place over 15 min in darkness at room temperature.

The brain slices were then mounted on slides, a mounting medium was applied, covered with a coverslip, and the staining was observed under a fluorescence microscope. The intensity of immunofluorescence was calculated using ImageJ software to assess the area of damage to dopamine neurons.

### 2.8 Data analysis

All statistical data are depicted as mea*n* ± S.E.M. Open field test data and immunofluorescence staining results were statistically analyzed using Student’s t-test. Analysis of variance (ANOVA) was used for statistical analysis of AIMs, rotarod test, gait analysis, spontaneous excitatory postsynaptic currents (sEPSCs), LTP, LTD, and dendritic spine density. In this study, all data analyses and graphing were performed using Prism 10.1.2 software.

## 3 Results

### 3.1 PD mouse model establishment

**Figure 2:**
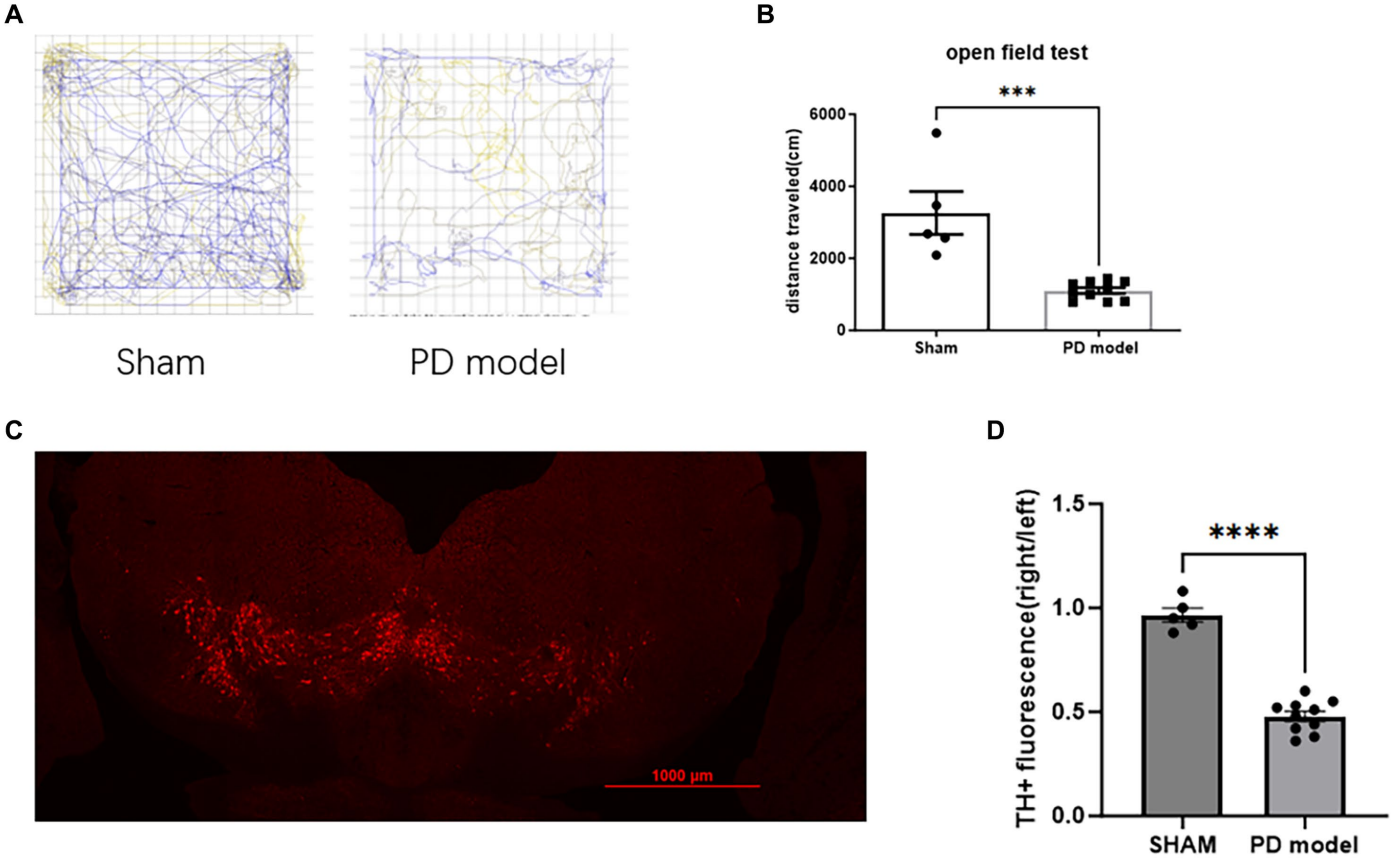
Assessment of PD modeling. **(A)** Representative movement trajectories of sham mice and PD mice model in the open field for 30 min. **(B)** Movement distance in the open field for 30 min. **(C)** Representative immunofluorescence labeling of TH in dopamine neurons within the SNc of mice with PD. **(D)** TH+ cells relative immunofluorescence density in the SNc. ***p < 0.001, ****p < 0.0001.

In the open field test, PD model mice showed a reduction in movement distance (Student’s *t*-test, *t* = 5.099, df = 13, *p* = 0.0002; Figures 2A,B), consistent with the bradykinesia symptoms of PD.

In PD mice model, the fluorescence intensity of TH+ cells in SNc on the 6-OHDA lesioned side was reduced (Student’s *t*-test, *t* = 11.49, df = 13, *p* < 0.0001; Figures 2C,D), indicating a depletion of DA neurons.

### 3.2 IRL790 treatment reduces AIMs

To investigate the therapeutic effect of the D3R antagonist IRL790 on LID, for 7 consecutive days, mice in the LID group and the IRL790 + LID group were administered L-dopa and benserazide. Additionally, mice in the IRL790 + LID group were given an intracranial injection of IRL790 into the striatum.

**Figure 3:**
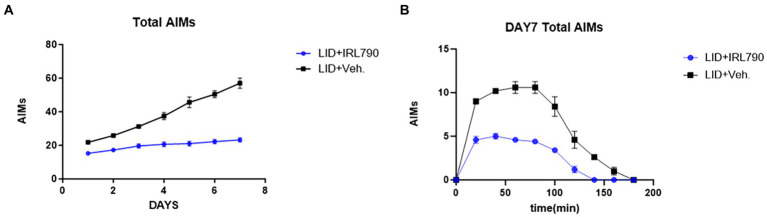
Total Abnormal Involuntary Movement Scores. **(A)** Daily total AIMs scores for the LID versus LID+IRL790 groups across days 1–7. **(B)** Total AIMs scores from 20 min to 160 min on day 7 for the LID and LID+IRL790 groups.

Mice in the LID+IRL790 group had lower AIMs scores than those in the LID group on days 1–7 [two-way ANOVA: time effect: *F*_(6, 56)_ = 41.31, *p* < 0.0001; treatment effect: *F*_(1, 56)_ = 400.9, *p* < 0.0001; time × treatment interaction: *F*_(6, 56)_ = 18.09, *p* < 0.0001; Tukey’s test, both *p* < 0.05; Figure 3A]. Moreover, within 20–140 min on day 7, the AIMs scores for mice in the LID+IRL790 group were lower than those in the LID group [two-way ANOVA: time effect: *F*_(9, 80)_ = 110.4, *p* < 0.0001; treatment effect: *F*_(1, 80)_ = 277.3, *p* < 0.0001; time × treatment interaction: *F*_(9, 80)_ = 13.73, *p* < 0.0001; Tukey’s test, both *p* < 0.05; Figure 3B]. Overall, the combined treatment with IRL790 significantly ameliorated motor disorders associated with LID.

### 3.3 IRL790 does not influence the benefit of L-dopa on a PD model

**Figure 4:**
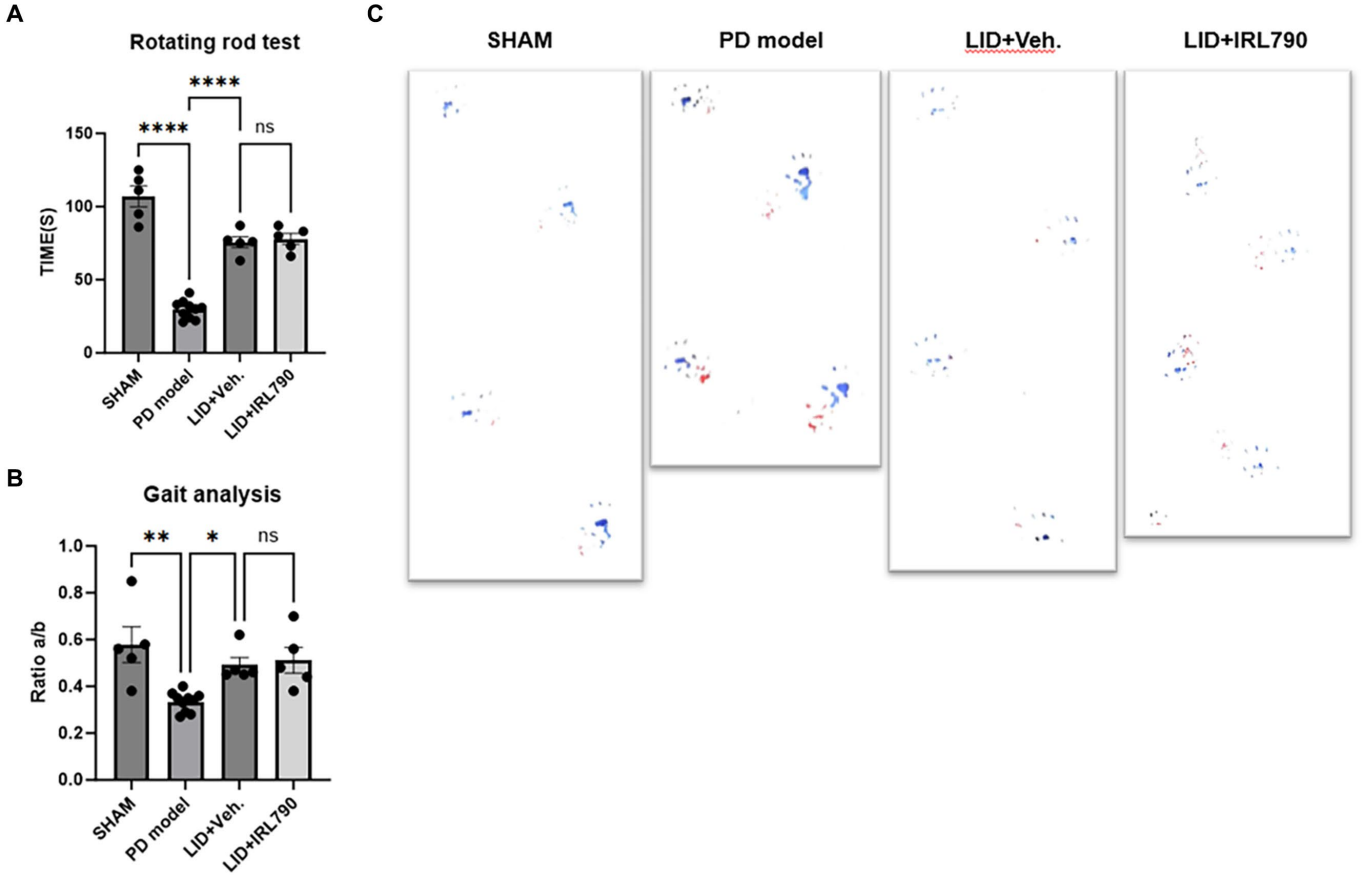
Rotarod test and gait analysis. **(A)** Duration of movement on the rotarod for mice in each group. **(B)** The ratio of the distance between the left and right hind paws **(A)** to the distance between steps of the left hind paw **(B)** (a/b ratio) for mice in each group. **(C)** Representative footprints from the gait analysis experiment for mice in each group. *p < 0.05, **p < 0.01, ****p < 0.0001.

The rotarod test and gait analysis are standard methods for evaluating motor abilities in cases of PD models. In the rotarod test, PD mice model remained on the rotarod for significantly shorter durations than the sham mice [one-way ANOVA, *F*_(3, 21)_ = 80.90, *p* < 0.0001; Tukey’s test, Sham vs. PD *p* < 0.0001; Figure 4A]. LID mice stay on the rotarod for longer periods compared to the PD mice model [one-way ANOVA, *F*_(3, 21)_ = 80.90, *p* < 0.0001; Tukey’s test, PD vs. LID *p* < 0.0001; Figure 4A]. The time spent on the rotarod by mice in the LID+IRL790 group showed no significant difference compared to LID mice [one-way ANOVA, *F*_(3, 21)_ = 80.90, *p* < 0.0001; Tukey’s test, LID vs. LID+IRL790 *p* = 0.9836; Figure 4A].

In the gait analysis, motor abilities were assessed using the ratio of the distance between the left and right hind paws (a) to the distance between steps of the left hind paw (b) (a/b ratio). The gait analysis score for PD mice model was lower than that for sham mice [one-way ANOVA, *F*_(3, 21)_ = 7.920, *p* = 0.001; Tukey’s test, Sham vs. PD *p* = 0.0013; Figure 4B]. The score for LID mice was higher than for PD mice model [one-way ANOVA, *F*_(3, 21)_ = 7.920, *p* = 0.001; Tukey’s test, PD vs. LID *p* = 0.0475; Figure 4B]. The score for mice in the LID+IRL790 group showed no significant difference compared to LID mice [one-way ANOVA, *F*_(3, 21)_ = 7.920, *p* = 0.001; Tukey’s test, LID vs. LID+IRL790 *p* = 0.9857; Figure 4B].

The motor abilities of LID mice were stronger than those of PD mice model, and the motor abilities of the LID+IRL790 group showed no difference compared to LID mice. This indicates that IRL790, while ameliorating the motor disorders induced by L-dopa, does not influence the benefit of L-dopa on PD.

### 3.4 IRL790 Improves abnormal functional correlates of synaptic plasticity in striatal D1+ MSNs of LID mice

It is currently believed that the pathogenesis of LID is closely related to synaptic plasticity changes in D1+ MSNs along the direct pathway of the striatum (Fieblinger et al., 2014). Therefore, we employed brain slices from D1-CRE mice that had been injected with rAAV-Ef1a-DIO-mCherry-WPRE-pA virus into the striatum to conduct electrophysiological recordings of striatal D1+ MSNs.

**Figure 5:**
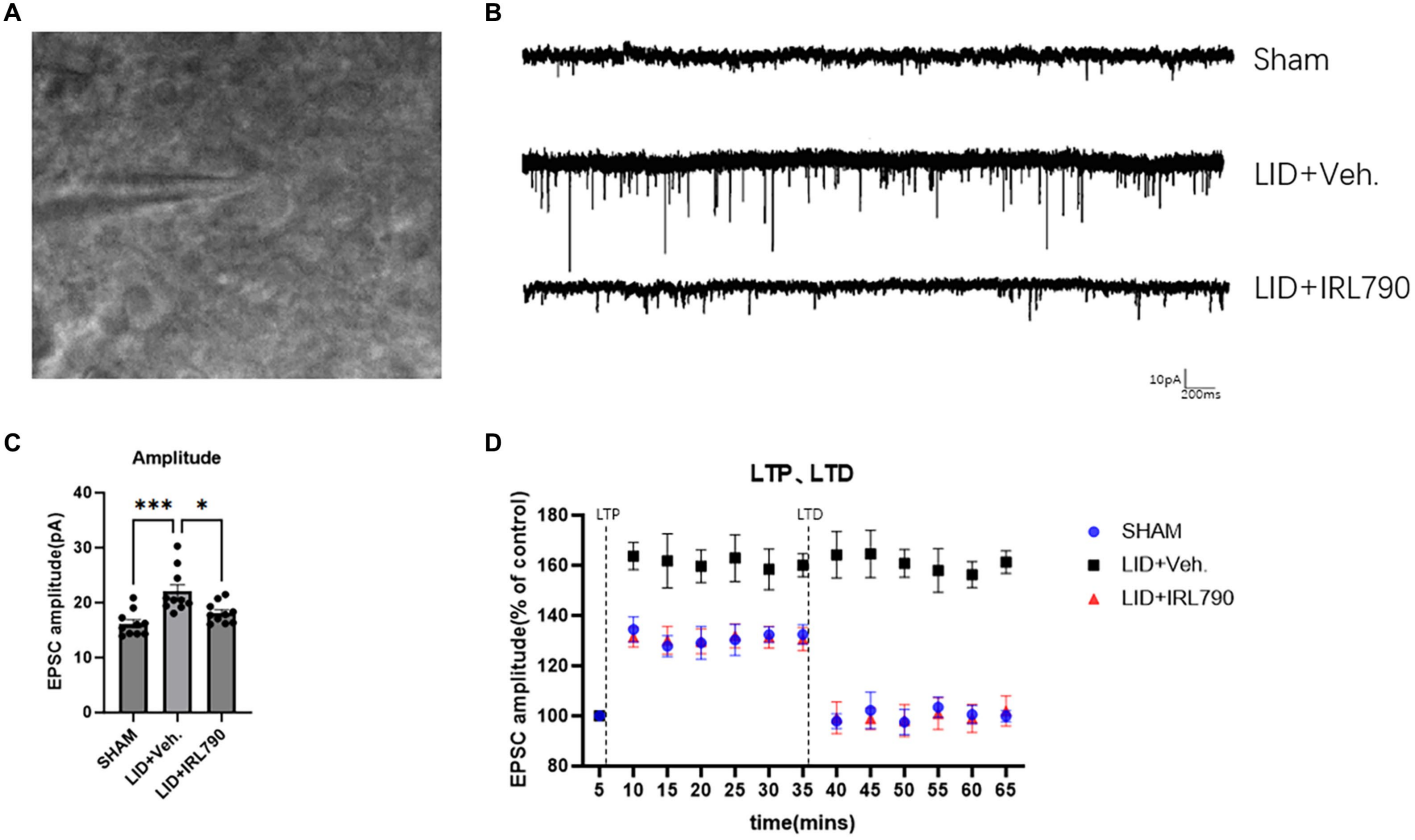
Patch-clamp electrophysiological recordings of D1 MSNs. **(A)** Representative images of cells clamped with a patch clamp. **(B)** Representative sEPSCs from mice in each group. **(C)** Amplitude of sEPSCs in mice from each group (each group *n* = 10 cells from 5 mice). **(D)** The enhanced magnitude of LTP induced (% of baseline) and the reduced magnitude of LTD (% of baseline) in mice from each group (each group *n* = 10 cells from 5 mice). *p < 0.05, ***p < 0.001.

In the recordings of sEPSCs in D1+ MSNs, LID mice exhibited higher current amplitudes than Sham mice [one-way ANOVA, *F*_(2, 27)_ = 10.77, *p* = 0.0004; Tukey’s test, Sham vs. LID *p* = 0.0003; Figures 5B,C]. In the LID+IRL790 group, the amplitude of sEPSCs was significantly reduced compared to the LID group when treated with a combination of L-dopa and IRL790 [one-way ANOVA, *F*_(2, 27)_ = 10.77, *p* = 0.0004; Tukey’s test, LID vs. LID+IRL790 *p* = 0.0136; Figures 5B,C]. The abnormally enhanced sEPSCs in LID mice could be a determining factor or an intermediate factor in the development of dyskinesia. The use of IRL790 improved the abnormally enhanced sEPSCs, trending toward the restoration of normal sEPSCs.

We utilized the spike-timing-dependent plasticity (STDP) paradigm (Fino et al., 2005) to induce and record LTP and LTD. The recordings revealed that, compared to sham mice, LID mice exhibited abnormally enhanced LTP [one-way ANOVA, *F*_(2, 27)_ = 141.3, *p* < 0.0001; Tukey’s test, Sham vs. LID *p* < 0.0001; Figure 5D]. In LID mice treated with a combination of IRL790, the abnormally enhanced LTP was restored [one-way ANOVA, *F*_(2, 27)_ = 141.3, *p* < 0.0001; Tukey’s test, LID vs. LID+IRL790 *p* < 0.0001; Figure 5D], with no significant difference observed in the enhancement of LTP compared to sham mice [one-way ANOVA, *F*_(2, 27)_ = 141.3, *p* < 0.0001; Tukey’s test, Sham vs. LID+IRL790 *p* = 0.6174; Figure 5D]. In the LTD recordings, LID mice were unable to induce LTD [Student’s *t*-test, *t* = 0.6839, df = 9, *p* = 0.5113; Figure 5D]. However, LTD was induced in LID mice treated with a combination of IRL790 (Student’s *t*-test, *t* = 9.492, df = 9, *p* < 0.0001; Figure 5D), and the reduction in LTD showed no significant difference compared to Sham mice [one-way ANOVA, *F*_(2, 27)_ = 67.86, *p* < 0.0001; Tukey’s test, Sham vs. LID+IRL790 *p* = 0.4428; Figure 5D].

Through the above electrophysiological recordings on D1+ MSNs, we found that IRL790 can reverse the pathological functional correlates of synaptic plasticity of D1+ MSNs, which helps to restore normal motor function.

### 3.5 IRL790 Improves abnormal structural correlates of synaptic plasticity in striatal D1+ MSNs of LID mice

Dendritic spines, serving as the structural foundation for synaptic plasticity, are categorized into three distinct types: mushroom, stubby, and thin. Mushroom and stubby spines are considered mature types, often involved in postsynaptic restructuring, while thin spines are regarded as immature (Suárez et al., 2014).

**Figure 6:**
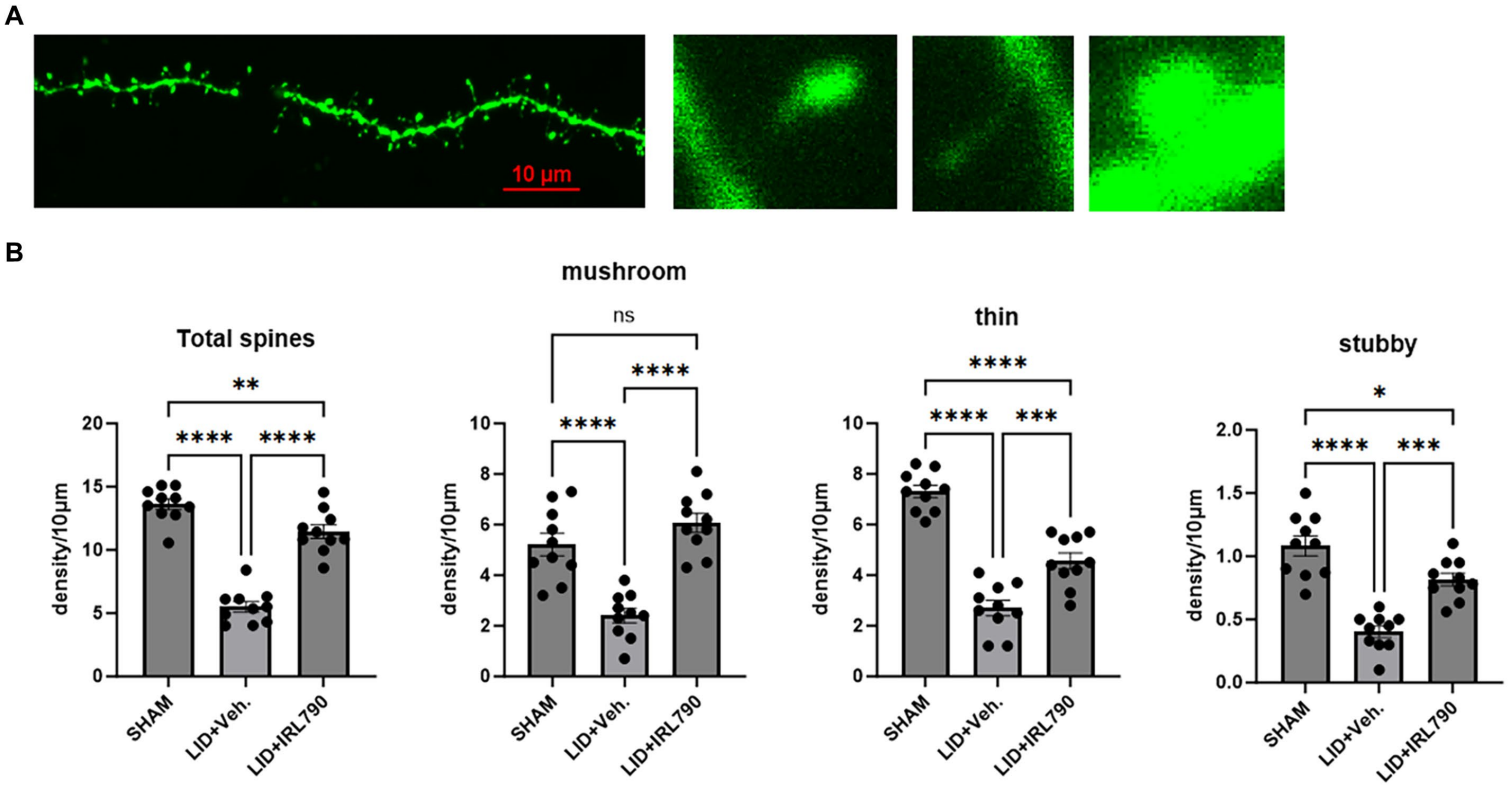
Dendritic spine density of D1 MSNs. **(A)** From left to right: representative dendritic spine morphology in sham mice; mushroom spine; thin spine; stubby spine. **(B)** Density of mushroom, thin, stubby, and total dendritic spines in mice from each group (each group *n* = 10 dendritic spines from 10 cells in 5 mice). *p < 0.05, **p < 0.01, ***p < 0.001, ****p < 0.0001.

Using Lucifer yellow intracellular staining for D1+ MSNs (Buhl and Schlote, 1987; Buhl and Lübke, 1989), we analyzed changes in the density of D1+ MSN dendritic spines. We found the total density of dendritic spines in LID mice was lower than that in sham mice [one-way ANOVA, *F*_(2, 27)_ = 81.43, *p* < 0.0001; Tukey’s test, Sham vs. LID *p* < 0.0001; Figure 6B], and combined treatment with IRL790 could improve the reduced total spine density [one-way ANOVA, *F*_(2, 27)_ = 81.43, *p* < 0.0001; Tukey’s test, LID vs. LID+IRL790 *p* < 0.0001; Figure 6B].Changes in dendritic spine density were observed in mushroom, thin and stubby spines. Mushroom, thin and stubby densities in LID mice were lower than in sham mice. Mice in the IRL790 + LID group showed higher densities of mushroom, thin and stubby dendritic spines compared to LID mice [one-way ANOVA: mushroom, *F*_(2, 27)_ = 26.34, *p* < 0.0001; Tukey’s test, Sham vs. LID *p* < 0.0001, LID vs. IRL790 + LID *p* < 0.0001; thin, *F*_(2, 27)_ = 62.73, *p* < 0.0001; Tukey’s test, Sham vs. LID *p* < 0.0001, LID vs. IRL790 + LID *p* = 0.0003; stubby, *F*_(2, 27)_ = 32.48, *p* < 0.0001; Tukey’s test, Sham vs. LID *p* < 0.0001, LID vs. IRL790 + LID *p* < 0.0001; Figure 6B]. Particularly, the mushroom spines, which are involved in postsynaptic structural remodeling, IRL790 + LID mice showed no significant difference compared to sham mice [one-way ANOVA, *F*_(2, 27)_ = 26.34, *p* < 0.0001; Tukey’s test, Sham vs. LID+IRL790 *p* = 0.2606; Figure 6B]. Therefore, it can be stated that IRL790 can reverse the pathological structural correlates of synaptic plasticity of D1+ MSNs in the striatum.

## 4 Discussion

Previous studies have demonstrated that D3R in the striatum are upregulated in L-dopa-treated 6-OHDA-lesioned rats (Bordet et al., 1997) and MPTP-treated primates (Bézard et al., 2003). Knockout of D3 receptors can attenuate levodopa-induced dyskinesias (LID) in mice (Solís et al., 2015). Treatment with L-dopa induced involuntary movements in non-Parkinsonian rodent models with overexpressed D3 receptors (Cote et al., 2014), and the application of D3R antagonists reduced dyskinetic behavior in experimental animals with LID (Kumar et al., 2009; Veselinović et al., 2013; Rice et al., 2015; Zhou et al., 2018; Waters et al., 2020). Research on D3R antagonists for the treatment of LID has primarily used intraperitoneal injections. Considering that D3R are expressed not only in the dStr but also in the nucleus accumbens, ventral pallidum, and the Islands of Calleja (Sokoloff et al., 1990), this study administered drugs intracranially to specifically target receptors in the dStr. The results indicate that mice receiving intracranial injections of IRL790 exhibited reduced dyskinetic behavior throughout the course of L-dopa injections. This suggests that IRL790 improves movement disorders by regulating receptors in the striatum.

Furthermore, previous research on the treatment of LID with D3R antagonists did not address whether these antagonists affect the therapeutic action of L-dopa on PD (Kumar et al., 2009; Veselinović et al., 2013; Rice et al., 2015; Zhou et al., 2018; Waters et al., 2020). Gait analysis and rotarod tests are primarily used to assess motor function in animal models (such as mice or rats), revealing the recovery process after injury, motor behavioral changes in disease models, and the impact of drugs on motor function (Mulherkar et al., 2013; Bhimani et al., 2017). Through gait analysis and rotarod tests, common experiments for assessing motor ability, we found no significant difference in motor ability between LID mice treated with L-dopa alone and those co-treated with IRL790. This indicates that IRL790 does not influence the benefit of L-dopa on PD while ameliorating LID.

The loss of synaptic plasticity in the striatum may lead to the pathologic storage of unnecessary motor information, resulting in the expression of abnormal movement patterns (Picconi et al., 2003). Previous studies have demonstrated that the pathogenesis of LID is related to changes in synaptic plasticity in D1+ MSNs within the direct pathway, including both structural and functional plasticity. Structurally, abnormalities in striatal D1+ MSNs, such as an increased number of dendritic spines and morphological changes, have been observed (Fieblinger et al., 2014). Functionally, there is abnormal and sustained LTP, with the disappearance of LTD in D1+ MSN within the direct pathway (Zhang et al., 2013). In our study, electrophysiological recordings of D1+ MSNs in LID mice showed higher sEPSCs and LTP compared to sham mice, with an inability to induce LTD. In dendritic spine density analysis, the D1+ MSNs of LID mice showed a significantly reduced spine density compared to sham mice. These results confirm the abnormal changes in synaptic plasticity of D1+ MSNs in the direct pathway within LID, in agreement with the conclusions of previous studies.

We investigated the changes in synaptic structure and functional plasticity of striatal D1+ MSNs in LID mice under the regulation of the D3R antagonist IRL790, to gain a deeper understanding of the mechanism behind D3R antagonist treatment for LID from the perspective of synaptic plasticity. Our experiments found that co-treatment with IRL790 normalized the abnormally increased LTP and the disappeared LTD in D1+ MSNs of LID mice, as well as normalized the abnormally increased sEPSCs, with no significant differences from the electrophysiological activity of sham mice. The co-treatment with IRL790 also restored the reduction in dendritic spines observed in LID, especially the significant increase in mushroom-shaped dendritic spines involved in post-synaptic structural remodeling, returning to levels observed in sham mice. These results confirm that IRL790 reversed the pathological functional and structural synaptic plasticity of D1+ MSNs in the direct pathway of LID mice. Therefore, it reduced the pathological storage of unnecessary motor information caused by abnormal synaptic plasticity, leading to an improvement in dyskinesia.

However, the findings of this study have to be seen in light of some limitations. The concentration of IRL790 we used, in addition to primarily acting on D3R, also exerts off-target activity on D2R, serotonergic receptors, sigma receptors, and norepinephrine transporters. Therefore, we can only confirm that IRL790 can improve the synaptic plasticity and dyskinesia of D1+ MSNs in LID mice by acting on the striatum, but cannot confirm that IRL790 plays a role by acting on the striatum D3R receptor. Among the receptors affected by IRL790, D3R and D2R are mainly expressed in the dorsal striatum and are most related to LID. Activation of D2R has been shown to inhibit motor behavior, and inhibition of D2R promotes motor behavior. Therefore, we speculated that striatal D3R is likely to be the target of IRL790 in the treatment of LID, which needs to be confirmed by further experiments. Meanwhile, our study only confirmed that IRL790 reversed pathological synaptic plasticity of D1+ MSN in LID mice. How IRL790 change the plasticity of D1+ MSN in the context of LID has not been studied. The specific mechanism remains to be explored.

In summary, this study confirms that the IRL790 modulates the abnormal synaptic plasticity of D1 MSNs to improve LID manifestations. This study revealed the important role of IRL790 in improving striatum abnormal synaptic plasticity in LID.

## Data availability statement

The original contributions presented in the study are included in the article/Supplementary material, further inquiries can be directed to the corresponding author.

## Ethics statement

The animal study was approved by the Animal Ethics Committee of Southern Medical University of China. The study was conducted in accordance with the local legislation and institutional requirements.

## Author contributions

XW: Conceptualization, Formal analysis, Investigation, Writing – original draft, Writing – review & editing. WZ: Writing – review & editing.
